# Qualitative and Quantitative Evaluation of Heat-Induced Changes in Polyphenols and Antioxidant Capacity in *Prunus domestica* L. By-products

**DOI:** 10.3390/molecules24163008

**Published:** 2019-08-19

**Authors:** Anna Michalska, Aneta Wojdyło, Joanna Majerska, Krzysztof Lech, Jessica Brzezowska

**Affiliations:** 1 Department of Fruit, Vegetable and Plant Nutraceutical Technology, the Faculty of Biotechnology and Food Science, Wrocław University of Environmental and Life Sciences, 51-630 Wrocław, Poland; 2 Institute of Agricultural Engineering, the Faculty of Life Sciences and Technology, Wrocław University of Environmental and Life Sciences, 51-630 Wrocław, Poland

**Keywords:** plum, waste products, drying, water activity, polyphenols, antioxidant capacity

## Abstract

Plum pomace, an agro-industrial waste product has received attention due to the worldwide popularity of plums. During convection, the content of flavan-3-ols decrease, except drying at 90 °C, whereas the content of i.e., chlorogenic, 3-*p*- and 4-*p*-coumaroylquinic acids, quercetin rutinoside, and galactoside was observed to increase along with the increase in process temperature. The highest content of all identified polyphenols was found in plum pomace powders obtained using a combination of convective at 90 °C and microwave vacuum drying (MVD) at 120 W, whereas the highest retention of the group consisted of phenolic acids, flavonols, and anthocyanins was noted when CD 70 °C/MVD 120 W was used, pointing to a strong influence of the type of polyphenols on their changes caused by drying. The correlations between TEAC ABTS and the sum of flavonoids (*r* = 0.634) and anthocyanins (*r* = 0.704) were established. The multiple regression analysis showed that polyphenol content was more strongly affected by drying time than by maximum temperature, whereas antioxidant capacity was more influenced by maximum temperature of sample than by drying time.

## 1. Introduction

Processing is an integral part of plant food production. One of its aims is to extend the shelf life of food products so that they can be offered to consumers throughout the year. Vegetable and fruit processing produce main products, by-products, and waste. In fruit and vegetable juice production, waste consists mainly of pomace, which is perishable. This is largely due to pomace containing water, which enables a rapid increase in microbiological contaminants. It is estimated that the moisture content in pomace may exceed 80% [1]. The processing industry struggles to effectively manage waste. In Poland, fruit (2 million tonnes) and vegetable (0.8 million tonnes) processing generates about 300–350 thousand tonnes of waste annually [2]. Therefore, various methods have been sought out to make use of pomace, mainly because it is easily available. Moreover, fruit pomace contains ca. 7% of total proteins, ca. 7% of sugars, ca. 6% of fibre, ca. 2% of pectins, and ca. 0.9% of organic acids; it is also rich in elements such as magnesium, iron, potassium, or calcium [3]. Fruit pomace is a rich source of polyphenolic compounds as well [4,5]. Oszmiański and Wojdyło [4] discovered that the polyphenol content in chokeberry pomace was over three times higher than in chokeberry juice. Last but not least, finding ways to manage pomace allows for reducing expenditures related to the transport and disposal of processing waste, and thus the minimization environmental risks.

Plum is ranked third in terms of fruit production volume in Poland (109.5 tonnes) [6] and, as such, is an important raw material in the food industry. It is available on the market not only in fresh form, but also as frozen or dried fruit, juices, jams, marmalades, and as an ingredient in alcoholic beverages. Dried plums are a rich source of polyphenolic compounds and have high antioxidant capacity far exceeding even that of apples, strawberries, oranges, or grapes [7]. They are also rich in vitamins A, C, and E, and β-carotene and various mineral compounds. Plum pomace that remains after making plum juice contains ca. 80% more polyphenolic compounds than the plum juice itself [7]. Thus, looking for new ways to use it in the food industry seems to be in line with the newest trends in food technology [8], especially when the addition of such products may increase the bioactive compounds in the food stuff, leading to an improvement of the health-related properties of products obtained. The preparation of such additives for use by the food industry requires its appropriate preparation; they must be easy to use and easily to handled. For these reasons, the drying of plum pomace might provide products in a powder form that can be easily applied to a broad range of products. Among the drying methods used for fruit pomace preservation, one of the most popular is convective drying (CD), considered as it is one of the cheapest; however, due to the presence of oxygen and relatively high temperatures applied, a strong degradation of bioactive compounds may occur, and so the quality of the products obtained might be relatively low [8]. On the other hand, microwave vacuum drying (MVD) was already used for fruit pomace dehydration, improving the quality of products obtained after drying. It should be noted that the lower the content of water during MVD, the higher the temperature of the samples that may lead to local burning of the samples. Thus, the application of this method should be designed according to the material properties [8]. Little has been said in the literature about how different drying methods affect the quality of dried plum pomace. Therefore, the aim of this study was to determine influence of the parameters of selected drying methods on the physical and chemical properties of plum pomace powders. Additionally, a correlation between the drying time, the maximum temperatures of plum pomace samples, and chemical properties of the plum pomace powders was measured using the Multiple Linear Regression Analysis.

## 2. Results

### 2.1. Properties of Plum Pomace Powders

#### 2.1.1. Drying Kinetics

**Figure 1:**
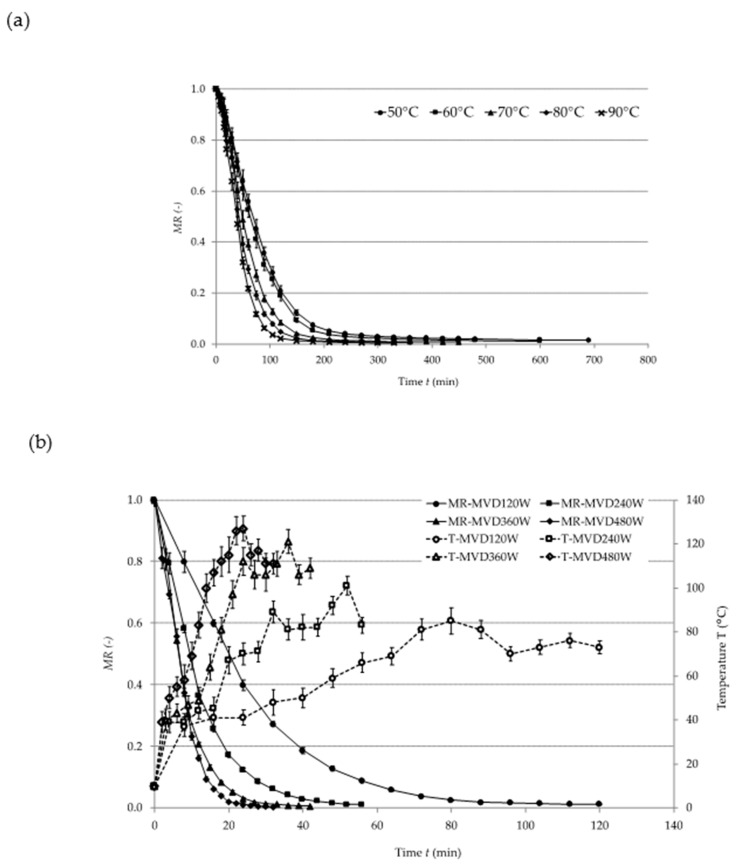

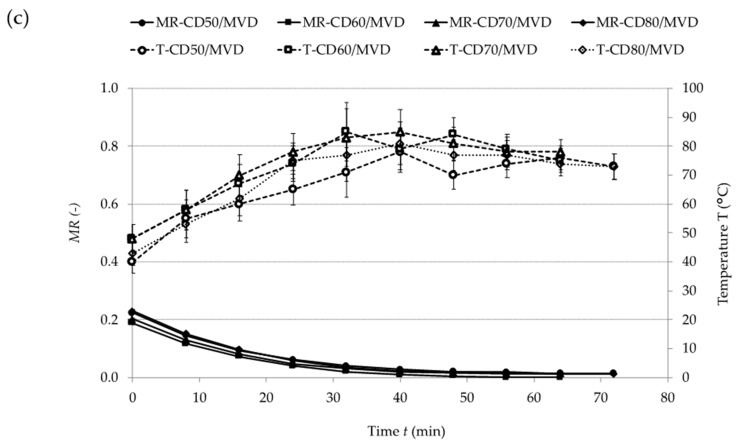
Drying kinetics of plum pomace dried by: (**a**) convective drying (CD), (**b**) microwave vacuum drying (MVD), (**c**) combined convective pre-drying and microwave vacuum finish-drying (CD/MVD). MR: moisture ratio.

Figure 1 presents drying curves indicating the reduction in the moisture content (MR) of plum pomace in relation to the drying time (t) during (a) CD, (b) MVD, and (c) CD/MVD. During CD, an increase in the temperature of hot air considerably shortened the drying time.

Similar observations were made during the convective drying of sour cherries [9], green gooseberries [10], and strawberries [11]. In the case of MVD, the increase in microwave power reduced the drying time [12]. During CD/MVD, no influence of the temperature of convective pre-drying on the time of microwave finish-drying was noted. This was probably due to the fact that the samples had similar moisture content after convective pre-drying (CPD).

The drying process was described by the modified Page model (Equation (1)):

(1) $$MR=A\times e^{\left(-k\times t^{n}\right)}+C$$

Table 1 presents the constants from Equation (1), the Root Mean Square Error (RMSE), and the coefficient of determination (R^2^).

Parameter A determines MR at *t* = 0 (beginning of drying by one of the applied methods). Parameters *k* and *n* determine the drying speed, and *C* is the asymptote value. Parameter *A* was 1 for CD and MVD, whereas it ranged between 0.1878 and 0.2290 for CD/MVD. This results from the fact that the samples were pre-dried up to a moisture content of 3.715 kg H_2_O∙kg^−1^ dry matter (dm). During CD, parameter *k* was 0.00218 for all temperatures applied, whereas parameter *n* rose along with the temperature. This means that parameter *n* had a stronger influence on the drying kinetics. MVD showed a similar pattern: parameter *k* was constant (0.0447), whereas parameter n increased along with the increase in microwave power. As for CD/MVD, parameter k remained the same (0.046), and parameter n varied only slightly due to the application of only one power level during finish-drying. For all studied samples, the RMSE was relatively low (0.0001–0.0346), while R^2^ reached values above 0.9867. This means that the applied models described the empirical points rather accurately.

Table 2 shows the drying times and the maximum temperatures of the samples. The longest drying time was noted for the freeze-dried control sample. CD at 50 °C lasted for 690 min. MVD at 480 W took the shortest time (32 min), but such microwave power caused considerable heating of the sample (T*_max_* = 127 °C). Similar observations were made during the drying of beetroots [13]. CD/MVD enabled the 2 up to 4-fold reduction in the drying time as compared to CD and prevented excessive heating of the samples (T*_max_* = 85 °C) thanks to applying the microwave power of 120 W. CD/MVD of the plum pomace was significantly shorter in comparison to whole-plum drying [14], which might be explained by different structure and moisture content in the pomace and thus different water binding capacity of the material. Similar conclusions were made in a study involving the drying of beetroots and carrots [15]. Taking the above into consideration, the design of the drying process should consider an initial structure of the materials.

#### 2.1.2. Physical Properties

The obtained plum pomace powders had moisture content between 0.014 and 0.067 kg H_2_O∙kg^−1^ dm (Table 2). Among the samples obtained, the highest moisture content was noted after FD. This might be connected with higher resistance to water vapor flow of plum pomace due to the presence of solids being higher when compared to the whole fruit. More solids in the material also influence mass transfer during the FD process that have a direct impact on the drying rate [16]. The final moisture content of the samples strongly depended on their maximum temperature of drying. Moisture levels below 0.02 kg H_2_O∙kg^−1^ dm were obtained during CD at 90 °C and MVD at 480 W. Lower temperatures and lower microwave power levels resulted in a moisture content of about 0.053 kg H_2_O∙kg^−1^ dm. Similar results were obtained for the drying of plums [14].

Water activity in the powders was below 0.35, which ensures microbiological security [17]. Water activity significantly depended on the moisture content of the material and reached the highest value (*a_w_*= 0.3465) for samples dried by CD at 50 °C, whose moisture content was 0.056 kg H_2_O∙kg^−1^ dm, and the lowest value (*a_w_*= 0.1690) for sampled dried by MVD at 480 W, with a moisture content of 0.01 kg H_2_O∙kg^−1^ dm. A similar relationship was found for blackcurrant [12].

True density was between 1.384 and 1.515 g∙cm^−3^ (Table 2) with no statistically significant differences between the samples. The factors determining true density are the composition of dry matter and the moisture content. The studied plum pomace powders had similar moisture content, so their true density was more strongly influenced by the composition of the dry matter, which does not considerably change during drying at a low temperature [18].

The freeze-dried samples had the lowest bulk density (0.467 g∙cm^−3^), much lower than the bulk density values identified after applying the other drying techniques (Table 2). This difference resulted from the structure of the obtained powders. During FD, the material forms’ amorphous structures [19], whereas CD and MVD lead to the formation of crystalline structures. The particles in a crystalline structure are heavier than those in an amorphous structure, so, within the same volume, the mass of a crystalline structure is greater than that of an amorphous structure [19], which translates into differences in bulk density. Due to the similar true density values of the studied samples, it was bulk density that had the main influence on the porosity of the obtained powders. The freeze-dried powders had the most porous structure (69.8%) because their bulk density was the lowest [12].

The color analysis of the plum pomace powders showed value of coordinate L* in the similar range for all samples. As in the case of blackcurrant pomace [12], the value of coordinate a* decreased along with the increase in temperature during CD. In samples dried by MVD at 120 W and 240 W, coordinate a* had similar values and decreased as the microwave power further increased, which was probably connected with the temperature of the samples. Coordinate a* values did not differ much in samples dried by CD/MVD either, or were more strongly determined by MVFD than CPD. The value of coordinate b* was the lowest in powders obtained after FD and was similar in all powders obtained using the other drying methods.

#### 2.1.3. Chemical Properties

The plum pomace is obtained after a juicing process, and thus it differs in terms of the content of biologically active components due to their higher share of the outer part of the plum fruit. The sum of all identified polyphenolic compounds in the plum pomace powders obtained using the selected drying methods ranged between 841 up to 1270 mg∙100 g^−1^ dm for samples obtained after convective drying at 80 °C and freeze-drying together with CD90/120 W, respectively. The dominant group of polyphenols consisted of flavan-3-ols (81.3 % of all analyzed polyphenols) that included polymeric procyanidins, (+)-catechin, and (−)-epicatechin (Table 3) [20]. Also, procyanidin C1 (trimer) and procyanidin B2 (dimer) were identified in the pomace powders. Both of these compounds were not found in plum juice powders [21], which indicates that they are mostly present in plum skin.

The second group of polyphenols present in plum pomace powders were phenolic acids accounting for over 12.5% of all polyphenols present in the studied pomace powders. Neochlorogenic acid was the most plentiful among polyphenolic acids identified in the analyzed plum pomace powders. This was in agreement with previous studies on plum products [20,22,23]. In the current work, it made up, on average, about 49% of all acids found in the dried products, regardless of the dehydration method.

The average content of neochlorogenic compound in pomace powders was almost five-times higher compared to its average content in the powders obtained from plum juice [21], which suggests its higher share in the outer parts of the fruit [24]. Other acids identified in the plum pomace powders included the following: chlorogenic acid (16%), caffeoylshikimic acid (14%), 3-*p*-coumaroylquinic acid (7%), 3-caffeoylshikimic acid (6%), 4-*O*-caffeoylquinic acid (4.3%), 4-*p*-coumaroylquinic acid (3%), and a phenolic acid derivative (2%). The content of chlorogenic acid was found to be almost two times as high compared to that in plum juice powders [21], which confirms a higher share of this compound in fruit skin [24]. Also, the fact that caffeoylshikimic acid and 4-*p*-coumaroylquinic acid were not identified in plum juices [21] suggests that these compounds are present in fruit skin and that the applied heating processes do not lead to their complete degradation. Thus, the retention of selected polyphenolic constituents can be obtained by an appropriate utilization of the fruit parts.

Another notable group of polyphenolic compounds present in dried plum pomace is the flavonoids (5.3%). The analyzed powders were found to contain 6 flavonoid compounds (Table 3), of which quercetin-3-*O*-rutinoside was the main compound constituting 62% of total flavonoids. This stands in contrast to the findings regarding the quercetin-3-*O*-rutinoside in powders obtained from plum juice [21] and even for whole plums [14] where the main flavonols were quercetin-*O*-galactoside and -xyloside, respectively. The results confirm that the content of selected polyphenolic compounds in dried plum products strictly depends on the fruit parts subjected to drying and may vary depending on both the share of those parts in the material being dried and process parameters. At the same time, there were over 50 % more total flavonoids in plum pomace powders than in plum powders, which may be attributable to a higher content of those compounds in fruit skin [20,22,25].

Similar to whole plum powders, anthocyanins were, in terms of content, the smallest polyphenolic group found in the analyzed pomace powders. In earlier studies, 6 anthocyanins were recognized in fresh plums [22,25]. In this study, only three anthocyanins were identified. As in fresh plums [26] and whole plum powders [14], cyanidin-3-*O*-rutinoside was the main compound accounting for about 61% of all anthocyanins. The second ranking compound was cyanidin-3-*O*-glucoside (34%), followed by peonidin-3-*O*-rutinoside, whose share was rather minor (5%).

The selected drying methods were found to have a significant influence on quantitative and qualitative changes in polyphenolic compounds. The highest content of all identified polyphenolic compounds was found in the powders obtained after the freeze-drying (Table 3). Since this method is considered to be time consuming and thus cost ineffective, other methods, i.e., CD, MVD, and the combination of CD and MVD were applied. The process temperatures and time (the availability of oxygen) during CD significantly influenced the presence of polyphenols. It was noted that the samples had the highest polyphenol content after being processed by convection at 90 °C. The polyphenol content was in that case around 25% higher than the lowest polyphenol content, which was found in samples dried at 80 °C. This was connected with the changes of polymeric procyanidins during drying, indicating that application of such a temperature might have led to a release of the compounds from more polymerized structures. Application of MVD at 120 W resulted in better retention of the sum of polyphenolic compounds when compared to the rest of the applied wattage of microwaves due to the lowest temperature applied during MVD. Similar to the whole plum drying [14], the combination of convective drying and microwave vacuum drying led to the higher content in a sum of identified polyphenols when compared to CV and MVD that was dependent on the structure of compounds. In general, changes during the drying of plum pomace by selected methods should have been considered in both aspects, i.e., among dominant group of polyphenols (flavan-3-ols) and among the rest of the compounds (phenolic acids, flavonols and anthocyanins) as the mechanism of their changes was significantly different. In detail, the content of individual polyphenols, i.e., chlorogenic acid, 3- and 4-*p*-coumaroylquinic acids, kaempferol derivative, quercetin-3-*O*-rutinoside, and quercetin-3-*O*-galactoside increased along with the temperature during CD (Table 3). This may have been due to the release of those compounds from other structures as a result of increased temperature [14] and/or the thermal treatment led to the formation of degradation products of chlorogenic acids, especially when the sucrose and high temperature of the process are concerned [27]. In the case of MVD, the biggest degradation of groups of these polyphenolic compounds was observed at 240 W microwave power. Thus, not only the temperature but also the processing time influenced the content of those constituents. The application of higher power levels (360 W and 480 W) led to a considerable reduction in polyphenols compared to 120 W. Therefore, the latter microwave power level was used in CD/MVD. The combined method (convective pre-drying at 70 °C and microwave vacuum finish-drying at 120 W) enabled obtaining the highest content of phenolic acids, flavonols, and anthocyanins in the analyzed pomace powders. Such an approach was in accord with Michalska et al. [14], where the application of the above combination of drying methods resulted in the highest content of all polyphenols in whole plum powders. Moreover, CD/MVD allowed shortening the drying time for over 10-fold relative to FD. The average content of polyphenols in the powders obtained using CD/MVD, regardless of process temperature, exceeded the average content of those compounds after CD by over 14% and after MVD by about 20%.

#### 2.1.4. Antioxidant Capacity

The antioxidant capacity of the studied powders was between 2.7 and 4.6 mmol Trolox∙100 g^−1^ dm for TEAC ABTS and between 2.7 and 4.5 mmol Trolox∙100 g^−1^ dm for FRAP (Table 3). The highest TEAC ABTS and FRAP values were observed for the freeze-dried samples, whereas the powders obtained using MVD at 120 W had the best ABTS^●+^ radical scavenging and ferric ion reducing ability of all samples obtained using CD, MVD, and CD/MVD. During CD, the TEAC ABTS values were observed to increase along with the temperature of the pomace samples and were higher by 23% for samples dried at 90 °C than for those dried at 50 °C. This might have been partially related to the earlier noted increase in the selected polyphenol compounds (Table 3). As the antioxidant capacity of polyphenols is determined by their chemical structure [28], it is probable that the above-mentioned compounds influenced the antioxidant capacity of the samples.

In addition, the relatively high temperatures applied during the drying of plum pomace might have triggered the formation of new compounds with antioxidant properties [14,22]. An inverse relationship was noted for blackcurrant pomace where TEAC ABTS values decreased (by 35 %) when temperature increased [29]. No correlation was reported between the sum of identified polyphenols and antioxidant capacity during applied drying methods, as the dominant were the flavan-3-ols. On the other hand, correlations between the sum of polyphenols and a sum of flavonoids (*r* = 0.604) was established, pointing to the influence of the structure of selected compounds on the ability to scavenge free radicals. During CD/MVD, the TEAC ABTS values were similar, reaching an average of ca. 2.8 mmol Trolox∙100 g^−1^ dm, which was the lowest average value for all analyzed drying methods. The highest FRAP values, just like the TEAC ABTS values, were recorded for freeze-dried powders (4.5 mmol Trolox∙100 g^−1^ dm). Among CD, MVD and CD/MVD the highest FRAP values were observed for samples dried by CD at 90 °C. Similar to TEAC ABTS values, the FRAP values increased along with temperature during CD. During MVD, the FRAP values increased at the microwave power levels between 120 W and 360 W, and then slightly decreased above this power range. As with TEAC ABTS values, the average FRAP values were the highest for MVD (3.44 mmol Trolox∙100 g^−1^ dm) relative to CD and CD/MVD. The lowest FRAP values were observed for powders obtained after CD/MVD.

#### 2.1.5. Multiple Linear Regression Analysis

Multiple Linear Regression Analysis was used to identify the influence of the drying time and the maximum temperature of the samples on the content of selected polyphenolic compounds and antioxidant capacity (Table 4). The multiple regression analysis carried out for CD showed a significant influence of independent variables on the content of phenolic acids and anthocyanins. For the sum of phenolic acids, the drying time was a significant factor (*p* = 0.033). This is probably due to the fact that those compounds are more easily affected by the presence of oxygen than by temperature (*p* = 0.0617) within the studied range (from 50 to 90 °C). Additionally, drying time had a slightly stronger impact on flavonoids (*p* = 0.0072) than the maximum temperature (*p* = 0.0094). During the MVD process, an increase of magnetrons’ power resulted in an increase of the samples’ temperature that influenced the duration of the process [30]. Shortening of the drying time preserved the flavonoids from the degradation to some extent. It was noted that during CD/MVD, the TEAC ABTS values were affected only by the maximum temperature (*p* = 0.0452); the higher the temperature the strongest degradation of compounds able to scavenge ABTS radicals.

## 3. Materials and Methods

### 3.1. Materials

The material under study was the pomace of plums cv. Valor, from which juice was extracted using a hydraulic press (SRSE, Warsaw, Poland). The initial moisture content was 3.72 kg∙kg^−1^ of dry matter (dm). The obtained plum pomace was dehydrated using selected drying methods.

### 3.2. Methods

#### 3.2.1. Drying methods

Freeze drying (FD) (ca. 60 g) was carried out in a freeze dryer OE-950 (Labor, MIM, Hungary) for 24 h. The temperature within the drying chamber was −60 °C, while the heating plates had a temperature of about 30 °C. Convective drying (CD) was performed in a convection dryer designed at the Institute of Agricultural Engineering (Wrocław University of Environmental and Life Sciences, Poland). Portions of plum pomace (ca. 60 g) were placed on sieve trays (Φ = 100 mm) and dried at temperatures 50 °C, 60 °C, 70 °C, 80 °C, and 90 °C with an air velocity of 0.8 m∙s^−1^. Microwave vacuum drying (MVD) was performed in a microwave vacuum dryer SM-200 (Plazmatronika, Wrocław, Poland) at the following magnetron power modes: 120 W, 240 W, 360 W and 480 W. Pomace samples (ca. 60 g) were placed in a cylindrical drying container (Φ = 180 mm, L = 270 mm) of organic glass at a reduced pressure (4−6 kPa) and the container was rotated at a speed of 6 rpm. Every time the mass of the pomace samples was measured, their temperature was measured with an infrared camera Flir i50 (Flir Systems AB, Sweden). Combined drying (CD/MVD) started with convective pre-drying (CPD) of the pomace samples (ca. 60 g for each temperature) at temperatures of 50 °C, 60 °C, 70 °C, 80 °C, and 90 °C. The samples were pre-dried until their moisture content reached about 0.75 kg∙kg^−1^ dm. This was followed by microwave vacuum finish-drying (MVFD) in the dryer SM-200 (Plazmatronika, Wrocław, Poland) at 120 W in order to prevent the material from overheating.

All drying experiments (CD, MVD and combined CD/MVD) were performed in duplicate (*n* = 2). The pomace was dried until its final moisture level was about 0.05 kg∙kg^−1^ dm. The dried plum pomace was pulverized by a grinder (Grinder MKM 6003c, Bosch GmbH, Germany) working at maximum speed for about 30 s. The so-obtained product was then analyzed.

#### 3.2.2. Physical properties

##### Moisture Content

The moisture content of the plum pomace powders was measured by the vacuum-oven method, using a vacuum oven SPT-200 (ZEAMiL, Horyzont, Cracow, Poland) at a temperature of 80 °C for 24 h. Dry basis was measured in triplicate (*n* = 3). The measurement of mass losses in the pomace samples during drying enabled the determination of drying kinetics expressed as moisture content reduction as a function of time (Equation (2)) [31]:

(2) $$MR=\frac{M_{i}}{M_{0}}$$

where

*MR*: moisture ratio
*M_i_*: current moisture content,
*M*_0_: initial moisture content.

##### Water Activity

Water activity (*a_w_*) in the plum pomace powders was measured in triplicate (*n* = 3) using an AQUA LAB DewPoint water activity meter (Washington State, USA) at a temperature of 25 °C ± 2.

##### True and Bulk Density

The true density of the plum pomace powders was measured with a HumiPyc TM/model 2 Gas Pycnometer (InstruQuest Inc., Coconut Creek, FL, USA) using argon (p = 220 kPa), and was calculated according to the following equation (Equation (3)):

(3) $$\rho_{t}=\frac{m}{V}$$

where

*m*: mass of the sample,
*V*: total sample volume.

Bulk density was measured using a graduated cylinder (10 cm^3^) and laboratory scales PS 4500 R2 (Radwag, Poland), and calculated according to the following equation (Equation (4)):

(4) $$\rho_{b}=\frac{m_{c}}{V_{c}}$$

where

*m_c_*: sample mass in cylinder,
*V_c_*: sample volume in cylinder.

True density and bulk density were measured in triplicate (*n* = 3).

##### Porosity

Porosity (ε) was measured according to the following equation (Equation (5)):

(5) $$\varepsilon =\left(1-\frac{\rho_{b}}{\rho_{t}}\right)\times 100\;\%$$

where

*ρ_b_*: bulk density,
*ρ_t_*: true density.

##### Color

The color of the plum pomace powders was determined by designating coordinates in the CIE L*a*b* color space (L-lightness, a*-red-green axis, b*-blue-yellow axis), using a Chroma Meter CR-400 (Minolta, Osaka, Japan) and the standard illuminant D65. The colors were measured in triplicate (*n* = 3).

#### 3.2.3. Chemical properties

##### Identification and Quantification of Polyphenols

The presence of polyphenolic compounds was identified by the LC-PDA-MS method using the Acquity Ultraperformance LC system (Waters Corp., Milford, USA; [9]). The polyphenol extracts were prepared according to the procedure described by Wojdyło et al. [9]. The results were evaluated using the MassLynx 4.0 ChromaLynx Application Manager software (Waters Corp., Milford, CT, USA). The quantification of polyphenolic compounds in plum pomace powders was performed by ultra-performance liquid chromatography (UPLC-PDA) (Waters Corp., Milford, USA). Calibration curves (0.05–5 mg∙mL^−1^; R^2^ = 0.9998) were made from procyanidins B2 and C1, neochlorogenic and chlorogenic acid, quercetin, isorhamnetin and kaempferol-3-*O*-glucoside, cyanidin-3-*O*-glucoside, cyanidin-3-*O*-rutinoside, and peonidin-3-*O*-glucoside as standards (Extrasynthese, Genay, France). Quercetin and kaempferol derivatives were expressed as quercetin-3-*O*-glucoside and kaempferol-3-*O*-glucoside, and coumaroylquinic and caffeoylshikimic acid derivatives as chlorogenic acid. Anthocyanins, flavonol glycosides, phenolic acids and flavan-3-ols were detected at wavelengths of 360, 320, and 280 nm, respectively. The analysis was carried out in duplicate and the results were expressed in mg∙100 g^−1^ dm.

##### Antioxidant Capacity

The antioxidant capacity of extracts (100 mg of samples in 1.7 mL of 80% aqueous methanol; *v*/*v*) was evaluated in in-vitro tests, i.e., the ABTS radical cation decolorization assay (Trolox equivalent antioxidant capacity determined using ABTS assay; TEAC ABTS) [32] and the FRAP (Ferric Reducing Antioxidant Potential) assay [33]. The analysis was performed in duplicate (*n* = 2) and results were presented as an average expressed in mmol Trolox equivalent∙100 g^−1^dm.

#### 3.2.4. Statistical analysis

One-way analysis of variance (ANOVA), the least significance test HSD Tukey (*p* ≤ 0.05), and the multiple regression analysis (*p* = 0.05) [34] were applied to compare the samples. Statistical analyses were performed using STATISTICA 10 (StatSoft, Tulsa, Ok, USA).

## 4. Conclusions

The results showed that physical properties of plum pomace powders obtained by applying selected drying methods were typical for such products. The applied drying methods and process parameters had a significant influence on the qualitative and quantitative changes in polyphenolic compounds and antioxidant capacity. Powders obtained using the CD 90 °C/MVD 120 W combined method followed by MVD 120 W were characterized by the highest content of polyphenols, which was at the comparable level to freeze-dried control samples. This was strongly connected with content of the dominant group of polyphenolics present in dried plum pomace, flavan-3-ols. The drying time applied during CD/MVD was not considerably longer than that applied during MVD at 120 W. However, when the other groups of polyphenols were concerned (phenolic acids, flavonols and anthocyanins), the highest retention of these compounds was noted after CD 70 °C/MVD 120 W, pointing to a strong influence of the drying methods and parameters applied on the selected groups of polyphenols present in plum pomace. Thus, the changes of the polyphenolics should be linked to specific groups of compounds. No correlation was found between the sum of all identified polyphenols and antioxidant capacity measured by TEAC ABTS and FRAP; however, correlations between TEAC ABTS and the sum of flavonoids (*r* = 0.634) and anthocyanins (*r* = 0.704) were recognized. The multiple regression analysis showed that the content of polyphenols was more strongly influenced by the drying time than the maximum temperature of the sample, and vice versa, that antioxidant capacity was more strongly affected by the maximum temperature of the sample than by the drying time that was connected with the properties of the compounds present in the plum pomace.

## Figures and Tables

**Table 1:** Drying models calculated using the equation: $\;MR=A\times e^{\left(-k\times t^{n}\right)}+C$.

Drying Method	Drying Conditions	Constants				Statistics	
		*A*	*k*	*n*	*c*	RMSE	R^2^
**CD**	**50 °C**	1	0.00218	1.38	0.0184	0.0066	0.9997
	**60 °C**	1	0.00218	1.40	0.0143	0.0062	0.9997
	**70 °C**	1	0.00218	1.49	0.0115	0.0058	0.9998
	**80 °C**	1	0.00218	1.54	0.0096	0.0161	0.9982
	**90 °C**	1	0.00218	1.60	0.0053	0.0089	0.9994
**MVD**	**120 W**	1	0.0447	0.97	0.0040	0.0346	0.9867
	**240 W**	1	0.0447	1.24	0.0130	0.0106	0.9987
	**360 W**	1	0.0447	1.46	0.0121	0.016	0.9973
	**480 W**	1	0.0447	1.49	0.0001	0.0206	0.9957
**CD/MVD**	**50 °C/120 W**	0.2230	0.046	1.09	0.0120	0.0001	0.9997
	**60 °C/120 W**	0.1878	0.046	1.12	0.0001	0.0026	0.9981
	**70 °C/120 W**	0.2016	0.046	1.12	0.0106	0.0012	0.9996
	**80 °C/120 W**	0.2290	0.046	1.10	0.0090	0.0024	0.9988
	**90 °C/120 W**	0.1990	0.046	1.13	0.0060	0.0013	0.9995

Abbreviations: CD: convective drying, MVD: microwave vacuum drying, CD/MVD: combined convective pre-drying and microwave vacuum finish-drying; RSME: root mean square error, R^2^: coefficient of determination.

**Table 2:** Physical properties of dried plum pomace obtained using selected drying methods.

Drying Method	Process Conditions	Drying Time (min)	Max Temperature (°C)	Moisture Content (kg H_2_O∙kg ^−1^ dm)	Water Activity (-)	True Density (g∙cm^−3^)	Bulk Density (g∙cm^−3^)	Porosity (%)	Color		
									L*	a*	b*
**FD**	**−60°C/30 °C**	1440 ± 20 ^a^*	-	0.067 ± 0.016 ^a^	0.1617 ± 0.007 ^j^	1.515 ± 0.028 ^a^	0.467 ± 0.04 ^b^	69.48 ± 0.7 ^a^	47.32 ± 0.16 ^i^	14.68 ± 0.24 ^a^	13.63 ± 0.26 ^h^
**CD**	**50 °C**	690 ± 15 ^b^	50 ± 2 ^g^	0.056 ± 0.001 ^b^	0.3465 ± 0.001 ^a^	1.455 ± 0.016 ^a^	0.656 ± 0.012 ^ab^	54.92 ± 1.51 ^ab^	47.50 ± 0.26 ^i^	12.40 ± 0.21 ^e^	16.58 ± 0.46 ^g^
	**60 °C**	600 ± 20 ^c^	60 ± 2 ^f^	0.045 ± 0.002 ^b^	0.2977 ± 0.001 ^e^	1.464 ± 0.025 ^a^	0.876 ± 0.030 ^a^	40.24 ± 3.47 ^b^	50.78 ± 0.34 ^e^	11.45 ± 0.04 ^f^	17.76 ± 0.11 ^cde^
	**70 °C**	450 ± 15 ^d^	70 ± 2 ^e^	0.031 ± 0.004 ^c^	0.2414 ± 0.001 ^g^	1.438 ± 0.021 ^a^	0.765 ± 0.138 ^ab^	46.89 ± 8.51 ^ab^	54.42 ± 0.37 ^a^	10.87 ± 0.16 ^g^	17.54 ± 0.21 ^def^
	**80 °C**	360 ± 15 ^e^	80 ± 2 ^d^	0.024 ± 0.002 ^dc^	0.2098 ± 0.002 ^h^	1.452 ± 0.026 ^a^	0.714 ± 0.014 ^ab^	50.80 ± 0.32 ^ab^	52.54 ± 0.13 ^b^	10.34 ± 0.04 ^h^	16.61 ± 0.21 ^g^
	**90 °C**	330 ± 10 ^f^	90 ± 2 ^c^	0.017 ± 0.004 ^d^	0.1833 ± 0.002 ^i^	1.407 ± 0.024 ^a^	0.749 ± 0.069 ^ab^	46.72 ± 6.21 ^b^	53.80 ± 0.26 ^a^	10.16 ± 0.12 ^h^	18.46 ± 0.36 ^bc^
**MVD**	**120 W**	120 ± 8 ^h^	85 ± 3 ^cd^	0.044 ± 0.002 ^b^	0.2935 ± 0.001 ^e^	1.433 ± 0.025 ^a^	0.761 ± 0.106 ^ab^	46.83 ± 8.66 ^ab^	48.58 ± 0.45 ^gh^	12.58 ± 0.24 ^d^	16.88 ± 0.37 ^fg^
	**240 W**	56 ± 4 ^i^	101 ± 4^b^	0.040 ± 0.008 ^b^	0.2845 ± 0.001 ^f^	1.438 ± 0.027 ^a^	0.805 ± 0.019 ^a^	43.98 ± 0.17 ^b^	49.02 ± 0.13 ^efg^	13.14 ± 0.19 ^bc^	17.07 ± 0.37 ^efg^
	**360 W**	42 ± 3 ^i^	121 ± 4 ^a^	0.021 ± 0.004 ^dc^	0.1917 ± 0.002 ^i^	1.465 ± 0.046 ^a^	0.790 ± 0.122 ^ab^	46.24 ± 5.94 ^b^	50.49 ± 0.39 ^e^	11.61 ± 0.19 ^f^	16.87 ± 0.19 ^fg^
	**480 W**	32 ± 2 ^i^	127 ± 5 ^a^	0.014 ± 0.001 ^e^	0.1690 ± 0.002 ^j^	1.469 ± 0.033 ^a^	0.775 ± 0.115 ^ab^	47.31 ± 6.18 ^ab^	51.70 ± 0.13 ^bc^	10.80 ± 0.24 ^g^	17.84 ± 0.43 ^cd^
**CD/MVD**	**50 °C /120 W**	222 ± 4 ^g^	78 ± 2 ^de^	0.051 ± 0.008 ^b^	0.3344 ± 0.001 ^b^	1.433 ± 0.040 ^a^	0.705 ± 0.010 ^ab^	51.09 ± 2.64 ^ab^	45.97 ± 0.57 ^j^	12.13 ± 0.04 ^e^	16.37 ±0.13 ^g^
	**60 °C/120 W**	139 ± 4 ^h^	85 ± 3 ^dc^	0.051 ± 0.002 ^b^	0.3184 ± 0.001 ^c^	1.457 ± 0.030^a^	0.693 ± 0.114^ab^	52.55 ± 6.45^ab^	50.55 ± 0.31^def^	12.36 ± 0.11^de^	18.34 ± 0.37^bcd^
	**70 °C/120 W**	131 ± 4 ^h^	85 ± 2 ^dc^	0.047 ± 0.007 ^b^	0.2939 ± 0.001 ^e^	1.395 ± 0.021 ^a^	0.659 ± 0.125 ^ab^	52.69 ± 9.96 ^ab^	48.73 ± 0.39 ^fgh^	13.44 ± 0.18 ^b^	19.33 ± 0.47 ^a^
	**80 °C/120 W**	132 ± 4 ^h^	81 ± 3 ^d^	0.046 ± 0.003 ^b^	0.3067 ± 0.001 ^d^	1.398 ± 0.017 ^a^	0.665 ± 0.096 ^ab^	52.36 ± 7.72 ^ab^	47.70 ± 0.64 ^i^	13.43 ± 0.21 ^b^	18.70 ± 0.256 ^ab^
	**90 °C/120 W**	122 ± 4 ^h^	90 ± 3 ^c^	0.052 ± 0.003 ^b^	0.3107 ± 0.001 ^dc^	1.384 ± 0.024^a^	0.790 ± 0.009^ab^	42.96 ± 0.75^b^	47.91 ± 0.51^hi^	12.63 ± 0.07^f^	17.32 ± 0.23^def^

FD: freeze-drying; CD: convective drying; MVD: microwave vacuum drying; CD/MVD: combined convective pre-drying and microwave vacuum finish-drying; *a–j—average values (±SD) with the same superscript letter within a column are not statistically different (ANOVA HSD Tukey’s test, *p* ≤ 0.05).

**Table 3:** Polyphenolic compounds (mg∙100 g^−1^ dm) and antioxidant capacity (mmol Trolox∙100 g^−1^ dm) of plum pomace powders obtained using selected drying methods (*n* = 3).

	FD	CD					MVD				CD/MVD				
		CD 50 °C	CD 60 °C	CD 70 °C	CD 80 °C	CD 90 °C	MVD 120	MVD 240	MVD 360	MVD 480	50 °C/120 W	60 °C/120 W	70 °C/120 W	80 °C/120 W	90 °C/120 W
**PROCYANIDINS**														
**Polymeric procyanidins**	680.5 ± 11.2 ^cde*^	693.6 ± 0.2 ^cde^	673.8 ± 6.2 ^de^	613.1 ± 27.7 ^ef^	476.9 ± 10.7 ^g^	769.8 ± 3.1 ^bc^	831.1 ± 31.6 ^ab^	726.6 ± 9.9 ^cd^	532.4 ± 12.3 ^fg^	676.3 ± 16.6 ^de^	651.8 ± 8.7 ^de^	690.5 ± 3.5 ^cde^	676.5 ± 6.6 ^de^	712.2 ± 9.4 ^cd^	889.8 ± 70.3 ^a^
**(+)-catechin**	148.1 ± 6.1 ^a^	108.5 ± 0.4 ^e^	109.3 ± 1.8 ^e^	114.1 ± 0.5 ^cde^	113.1 ± 7.1 ^cde^	109 ± 6.7 ^e^	127.5 ± 2.7 ^bc^	131.9 ± 2.5 ^b^	136.6 ± 4.7 ^ab^	125.1 ± 3.3 ^bcd^	116.5 ± 4.9 ^cde^	110.5 ± 2.0 ^de^	111.2 ± 2.2 ^de^	106.4 ± 2.1 ^e^	115.7 ± 1 ^cde^
**(-)-epicatechin**	18.3 ± 1.4 ^a^	14.2 ± 1.1 ^abc^	12.4 ± 1.9 ^c^	13.7 ± 1.2 ^bc^	10.6 ± 1.2 ^cde^	6.5 ± 0.4 ^e^	13.7 ± 0.8 ^bc^	16.8 ± 1 ^ab^	10.1 ± 0.1 ^cde^	6.9 ± 0.4 ^de^	12.8 ± 0.7 ^bc^	11.1 ± 0.2 ^cd^	10.5 ± 0.4 ^cde^	10.2 ± 0.1 ^cde^	12.4 ± 2.2 ^c^
**Trimer**	36.1 ± 1.7 ^a^	29.9 ± 0.1 ^c^	30.1 ± 0.1 ^c^	31.5 ± 0.9 ^bc^	29.9 ± 0.1 ^c^	29.3 ± 1.2 ^c^	33.8 ±0.9 ^ab^	28.5 ± 0.5 ^c^	30.1 ± 1.0 ^c^	31.6 ± 0.1 ^bc^	29.1 ± 0.8 ^c^	29.9 ± 0.5 ^c^	33.7 ± 0.8 ^ab^	29.1 ± 0.3 ^c^	28.7 ± 0.0 ^c^
**Dimer**	20.6 ± 1.1 ^a^	15.6 ± 0.6 ^cdef^	15.7 ± 0.3 ^cdef^	15.9 ± 0.0 ^cdef^	15.5 ± 0.1 ^def^	14.1 ± 0.6 ^ef^	18.3 ± 0.7 ^b^	15.5 ± 0.1 ^def^	16.2 ± 0.8 ^cde^	17.1 ± 0.0 ^bcd^	14.9 ± 0.4 ^ef^	15.5 ± 0.3 ^def^	17.6 ± 0.1 ^bc^	14.7 ± 0.1 ^ef^	14.1 ± 0.0 ^f^
**PHENOLIC ACIDS**														
**Neochlorogenic acid**	137.2 ± 0.8 ^a^	53.1 ± 1.1 ^f^	54.9 ± 0.4 ^f^	66.7 ± 0.1 ^d^	65.4 ± 0.2 ^de^	61.2 ± 0.6 ^e^	47.8 ± 0.4 ^gh^	38.4 ± 0.7 ^i^	45.3 ± 3.6 ^h^	49.9 ± 0.5 ^fgh^	52.2 ± 2.2 ^fg^	69.3 ± 0.8 ^d^	96.7 ± 0.8 ^b^	82.1 ± 1.0 ^c^	69.2 ± 0.5 ^d^
**Chlorogenic acid**	33.2 ± 0.2 ^a^	16.6 ± 0.2 ^ef^	17.7 ± 0.2 ^e^	24.3 ± 0.3 ^d^	26.3 ± 0.4 ^c^	26.4 ± 0.1 ^c^	12.6 ± 0.1 ^g^	9.8 ± 0.4 ^h^	13.8 ± 0.0 ^g^	15.5 ± 0.7 ^f^	16.9 ± 0.1 ^e^	23.3 ± 0.2 ^d^	32.5 ± 0.7 ^a^	32.3 ± 0.3 ^a^	30.6 ± 0.1 ^b^
**3-*p*-Coumaroylquinic acid**	15.1 ± 0.2 ^a^	6.6 ± 0.1 ^gh^	7.2 ± 0.2 ^h^	9.4 ± 0.1 ^f^	10.2 ± 0.2 ^e^	11.4 ± 0.1 ^cd^	6.2 ± 0.2 ^h^	5.1 ± 0.1 ^i^	6.2 ± 0.5 ^h^	6.8 ± 0.0 ^gh^	6.9 ± 0.2 ^gh^	8.9 ± 0.2 ^f^	12.5 ± 0.2 ^b^	11.9 ± 0.1 ^cd^	11.2 ± 0.0 ^d^
**3-Caffeoylshikimic acid**	13.6 ± 0.1 ^a^	7.1 ± 0.3 ^def^	6.8 ± 0.1 ^fgh^	6.8 ± 0.1 ^fg^	6.1 ± 0.1 ^hi^	4.7 ± 0.0 ^j^	7.8 ± 0.1 ^cd^	5.8 ± 0.2 ^i^	6.3 ± 0.3 ^ghi^	7.1 ± 0.0 ^ef^	7.1 ± 0.3 ^ef^	8.4 ± 0.1 ^c^	10.8 ± 0.3 ^b^	7.7 ± 0.1 ^cde^	6.6 ± 0.1 ^fgh^
**Derivative of phenolic acid**	2.8 ± 0.3 ^e^	1.6 ± 0.1 ^gh^	1.7 ± 0.1 ^fg^	2.8 ± 0.1 ^e^	3.4 ± 0.2 ^cd^	5.1 ± 0.1 ^a^	1.3 ± 0.1 ^gh^	1.2 ± 0.0 ^h^	1.4 ± 0.0 ^gh^	1.6 ± 0.0 ^gh^	1.7 ± 0.1 ^fg^	2.1 ± 0.1 ^f^	3.1 ± 0.1 ^de^	3.7 ± 0.1 ^c^	4.2 ± 0.1 ^b^
**4-*O*-Caffeoylquinic acid**	5.3 ± 0.4 ^e^	3.8 ± 0.2 ^cde^	3.8 ± 0.1 ^cde^	4.1 ± 0.1 ^cd^	3.9 ± 0.1 ^cd^	3.5 ± 0.0 ^def^	4.1 ± 0.1 ^cd^	3.1 ± 0.0 ^f^	3.3 ± 0.2 ^ef^	3.7 ± 0.1 ^cde^	3.6 ± 0.1 ^def^	3.9 ± 0.1 ^cde^	4.7 ± 0.1 ^ab^	4.2 ± 0.0 ^c^	4.2 ± 0.0 ^bc^
**Caffeoylshikimic acid**	35.5 ± 0.2 ^a^	16.8 ± 0.9 ^f^	15.8 ± 0.2 ^fg^	15.8 ± 0.2 ^fg^	14.1 ± 0.2 ^h^	10.9 ± 0.2 ^i^	19.2 ± 0.2 ^cd^	14.1 ± 0.3 ^gh^	15.4 ± 0.9 ^fgh^	17.1 ± 0.1 ^ef^	17.1 ± 0.6 ^f^	20.6 ± 0.1 ^c^	26.5 ± 0.6 ^b^	18.7 ± 0.1 ^de^	15.7 ± 0.2 ^fgh^
**4-*p*-Coumaroylquinic acid**	5.3 ± 0.4 ^b^	3.2 ± 0.1 ^de^	3.4 ± 0.1 ^d^	4.6 ± 0.1 ^c^	5.1 ± 0.1 ^bc^	5.1 ± 0.1 ^cb^	2.9 ± 0.0 ^de^	2.1 ± 0.1 ^f^	2.7 ± 0.0 ^ef^	3.2 ± 0.2 ^de^	3.5 ± 0.1 ^d^	4.7 ± 0.1 ^c^	6.4 ± 0.2 ^a^	6.2 ± 0.0 ^a^	5.6 ± 0.1 ^b^
**FLAVONOIDS**														
**Isorhamnetin-3-*O*-glucoside**	10.7 ± 0.1 ^a^	7.2 ± 0.1 ^f^	8.3 ± 0.4 ^cde^	8.2 ± 0.0 ^cde^	8.5 ± 0.3 ^cde^	8.7 ± 0.0 ^bcd^	8.9 ± 0.1 ^bc^	7.5 ± 0.3 ^ef^	7.8 ± 0.6 ^def^	8.5 ± 0.0 ^cd^	7.8 ± 0.3 ^def^	8.1 ± 0.1 ^cdef^	9.6 ± 0.3 ^b^	8.2 ± 0.0 ^cdef^	8.3 ± 0.1 ^cde^
**Quercetin-3-*O*-rutinoside**	47.4 ± 0.3 ^a^	29.6 ± 0.2 ^g^	34.1 ± 1.7 ^cdef^	34.7 ± 0.1 ^cdef^	35.2 ± 0.8 ^cde^	36.1 ± 0.7 ^cd^	36.9 ± 0.3 ^bc^	30.4 ± 0.8 ^g^	31.5 ± 2.1 ^fg^	34.1 ± 0.0 ^cdef^	31.6 ± 1.3 ^efg^	33.2 ± 0.6 ^defg^	39.7 ± 1.0 ^b^	34.8 ± 0.1 ^cdef^	35.8 ± 0.3 ^cd^
**Quercetin-3-*O*-galactoside**	7.4 ± 0.1 ^a^	4.7 ± 0.0 ^e^	5.3 ± 0.3 ^cde^	5.3 ± 0.0 ^cde^	5.6 ± 0.2 ^cd^	5.8 ± 0.2 ^bc^	5.8 ± 0.1 ^bc^	4.9 ± 0.1 ^de^	5.1 ± 0.3 ^cde^	5.5 ± 0.2 ^cde^	5.1 ± 0.4 ^cde^	5.2 ± 0.3 ^cde^	6.4 ± 0.1 ^b^	5.5 ± 0.1 ^cde^	5.8 ± 0.02 ^bc^
**Quercetin-3-*O*-xyloside**	7.4 ± 0.1 ^a^	5.1 ± 0.0 ^ghi^	5.6 ± 0.1 ^fgh^	5.3 ± 0.1 ^ghi^	5.1 ± 0.3 ^hi^	5.2 ± 0.2 ^ghi^	7.1 ± 0.2 ^bc^	6.4 ± 0.1 ^cd^	6.1 ± 0.4 ^def^	6.3 ± 0.1 ^cde^	5.4 ± 0.1 ^fghi^	5.2 ± 0.1 ^fghi^	5.8 ± 0.2 ^defg^	4.8 ± 0.2 ^i^	5.6 ± 0.0 ^efgh^
**Quercetin-3-*O*-glucoside**	0.7 ± 0.1 ^a^	0.5 ± 0.1 ^a^	0.5 ± 0.1 ^a^	0.5 ± 0.0 ^a^	0.5 ± 0.0 ^a^	0.5 ± 0.2 ^a^	0.6 ± 0.1 ^a^	0.5 ± 0.0 ^a^	0.6 ± 0.1 ^a^	0.6 ± 0.1 ^a^	0.5 ± 0.1 ^a^	0.6 ± 0.1 ^a^	0.6 ± 0.1 ^a^	0.5 ± 0.1 ^a^	0.5 ± 0.1 ^a^
**Derivatives of kaempferol**	1.5 ± 0.1 ^ab^	1.3 ± 0.1 ^bcd^	1.2 ± 0.0 ^bcde^	1.1 ± 0.0 ^def^	1.1 ± 0.1 ^cdef^	0.9 ± 0.1 ^fg^	1.4 ± 0.1 ^abc^	0.9 ± 0.0 ^efg^	1.1 ± 0.1 ^defg^	1.31 ± 0.0 ^bcd^	1.2 ± 0.0 ^bcde^	1.5 ± 0.1 ^ab^	1.7 ± 0.0 ^a^	1.1 ± 0.1 ^def^	0.8 ± 0.0 ^g^
**ANTHOCYANINS**														
**Cyanidin-3-*O*-glucoside**	14.2 ± 0.1 ^a^	2.7 ± 0.1 ^d^	2.4 ± 0.1 ^def^	2.3 ± 0.1 ^ef^	1.4 ± 0.0 ^g^	0.8 ± 0.1 ^h^	3.2 ± 0.0 ^c^	2.2 ± 0.0 ^f^	2.5 ± 0.0 ^def^	2.6 ± 0.0 ^de^	2.4 ± 0.0 ^ef^	3.4 ± 0.1 ^c^	4.5 ± 0.21 ^b^	2.4 ± 0.1 ^def^	1.2 ± 0.1 ^g^
**Cyanidin-3-*O*-rutinoside**	26.6 ± 0.1 ^a^	4.7 ± 0.3 ^d^	4.3 ± 0.3 ^de^	4.1 ± 0.0 ^de^	2.8 ± 0.1 ^g^	1.6 ± 0.0 ^h^	5.5 ± 0.1 ^c^	3.5 ± 0.0 ^f^	4.3 ± 0.3 ^de^	4.1 ± 0.1 ^ef^	4.1 ± 0.2 ^ed^	5.7 ± 0.2 ^c^	8.3 ± 0.01 ^b^	4.67 ± 0.1 ^de^	2.6 ± 0.2 ^g^
**Peonidin-3-*O*-rutinoside**	1.6 ± 0.1 ^a^	0.5 ± 0.0 ^c^	0.4 ± 0.1 ^cde^	0.4 ± 0.1 ^cde^	0.3 ± 0.1 ^de^	0.2 ± 0.0 ^e^	0.5 ± 0.0 ^cd^	0.3 ± 0.1 ^cde^	0.4 ± 0.0 ^cde^	0.4 ± 0.0 ^cde^	0.4 ± 0.0 ^cde^	0.5 ± 0.0 ^dc^	0.8 ± 0.01 ^b^	0.42 ± 0.0 ^cde^	0.2 ± 0.1 ^e^
**Sum of polyphenols**	1269.2 ± 1.1 ^a^	1027.2 ± 3.6 ^de^	1014.8 ± 8.4 ^de^	984.9 ± 27.2 ^e^	841.2 ± 0.5 ^f^	1117 ± 5.7 ^bc^	1196.5 ± 33.9 ^ab^	1056.1 ± 8.1 ^cde^	879.3 ± 9.6 ^f^	1025.5 ± 18.4 ^de^	992.5 ± 7.1 ^e^	1062.3 ± 0.2 ^cde^	1120.4 ± 2.4 ^bc^	1101.9 ± 9.1 ^cd^	1268.9 ± 67.8 ^a^
**FRAP**	4.5 ± 0.1 ^a^	2.9 ± 0.1 ^e^	2.9 ± 0.0 ^de^	3.1 ± 0.3 ^cde^	3.2 ± 0.1 ^bcde^	3.6 ± 0.1 ^b^	3.2 ± 0.0 ^bcde^	3.4 ± 0.2 ^bcd^	3.6 ± 0.3 ^b^	3.5 ± 0.1 ^bc^	2.7 ± 0.0 ^e^	2.7 ± 0.06 ^e^	2.8 ± 0.01 ^e^	2.8 ± 0.1 ^e^	2.8 ± 0.0 ^e^
**TEAC ABTS**	4.6 ± 0.1 ^a^	2.7 ± 0.0 ^g^	2.9 ± 0.2 ^def^	3.0 ± 0.2 ^cdef^	3.1 ± 0.1 ^cde^	3.5 ± 0.0 ^b^	3.2 ± 0.1 ^bcd^	3.6 ± 0.0 ^b^	3.4 ± 0.1 ^c^	3.1 ± 0.0 ^cd^	2.8 ± 0.1 ^efg^	2.7 ± 0.01 ^fg^	2.7 ± 0.02 ^fg^	2.8 ± 0.1 ^fg^	2.8 ± 0.1 ^defg^

FD: freeze-drying; CD: convective drying; MVD: microwave vacuum drying; CD/MVD: combined convective pre-drying and microwave vacuum finish-drying; TEAC ABTS: Trolox equivalent antioxidant capacity by determined using ABTS assay; FRAP: Ferric Reducing Antioxidant Potential; *a–j—average values (±SD) with the same superscript letter within a raw are not statistically different (ANOVA HSD Tukey’s test, *p* ≤ 0.05).

**Table 4:** Multiple linear regression analysis results for polyphenolic compounds and antioxidant capacity of plum pomace powders.

		CD						MVD						CD/MVD					
**Flavan-3-ols**	**Model**	**R^2^ = 0.4118**		**F_(2.2)_ = 2.400**	***p* < 0.294**	**RMSE = 81.412**		**R^2^ = 0.0001**		**F_(2.1)_ = 0.958**	***p* < 0.586**	**RMSE = 126.7**		**R^2^ = 0.7968**		**F_(2.2)_ = 8.842**	***p* < 0.102**	**RMSE = 42.94**	
	**Variables**	**β**	**SH_β_**	**B**	**SH_B_**	**T**	**p**	**β**	**SH_β_**	**B**	**SH_B_**	**T**	**p**	**β**	**SH_β_**	**B**	**SH_B_**	**T**	**p**
	**Constant**			2407.4	1509.6	−1.59	0.2518			468.75	1405.3	0.33	0.7950			2740.5	478.48	5.73	0.0292
	**Drying time**	4.11	1.89	2.818	1.29	2.18	0.1613	1.06	1.50	3.33	4.75	0.70	0.6104	−0.76	0.24	−1.75	0.55	−3.21	0.0851
	**Temp. max**	3.94	1.89	26.442	12.69	2.08	0.1725	0.27	1.50	1.93	10.60	0.18	0.8856	−0.84	0.24	−19.59	5.53	−3.54	0.0713
**Polymeric procyanidins**	**Model**	**R^2^ = 0.4684**		**F_(2.2)_ = 2.763**	***p* < 0.266**	**RMSE = 79.905**		**R^2^ = 0.0001**		**F_(2.1)_ = 0.859**	***p* < 0.607**	**RMSE = 130.5**		**R^2^ = 0.8403**		**F_(2.2)_ = 11.52**	***p* < 0.080**	**RMSE =3 8.02**	
	**Variables**	**β**	**SH_β_**	**B**	**SH_B_**	**T**	**p**	**β**	**SH_β_**	**B**	**SH_B_**	**T**	**p**	**β**	**SH_β_**	**B**	**SH_B_**	**T**	**p**
	**Constant**			2770.11	1481.4	−1.87	0.2024			193.84	1446.9	0.13	0.9152			2574.62	423.63	6.08	0.0260
	**Drying time**	4.21	1.80	2.98	1.27	2.34	0.1438	1.12	1.56	3.53	4.89	0.72	0.6018	−0.78	0.21	−1.80	0.48	−3.72	0.0651
	**Temp. max**	4.06	1.80	28.13	12.45	2.26	0.1523	0.37	1.56	2.61	10.91	0.24	0.8506	−0.83	0.21	-19.53	4.90	−3.99	0.0575
**Phenolic acids**	**Model**	**R^2^ = 0.9499**		**F_(2.2)_ = 38.96**	***p* < 0.0252**	**RMSE = 2.827**		**R^2^ = 0.7584**		**F_(2.1)_ = 5.709**	***p* < 0.284**	**RMSE = 5.517**		**R^2^ = 0.3695**		**F_(2.2)_ = 2.172**	***p* < 0.315**	**RMSE = 24.57**	
	**Variables**	**β**	**SH_β_**	**B**	**SH_B_**	**T**	**p**	**β**	**SH_β_**	**B**	**SH_B_**	**T**	**p**	**β**	**SH_β_**	**B**	**SH_B_**	**T**	**p**
	**Constant**			359.222	52.41	6.85	0.0206			110.473	61.17	−1.81	0.3219			9.37	277.09	0.03	0.9761
	**Drying time**	−2.96	0.55	−0.241	0.04	−5.37	0.033	2.391	0.73	0.678	0.21	3.28	0.1882	−0.656	0.42	−0.5	0.32	−1.58	0.2548
	**Temp. max**	−2.115	0.55	−1.69	0.44	-3.84	0.0617	2.43	0.73	1.538	0.46	3.34	0.1854	0.347	0.42	2.675	3.2	0.84	0.4913
**Flavonoids**	**Model**	**R^2^ = 0.4643**		**F_(2.2)_ = 2.734**	**p < 0.268**	**RMSE = 2.480**		**R^2^ = 0.9997**		**F_(2.1)_= 4478.8**	***p* < 0.011**	**RMSE = 0.0826**		**R^2^ = 0.0001**		**F_(2.2)_ = 0.645**	***p* < 0.608**	**RMSE = 5.15**	
	**Variables**	**β**	**SH_β_**	**B**	**SH_B_**	**T**	**p**	**β**	**SH_β_**	**B**	**SH_B_**	**T**	**p**	**β**	**SH_β_**	**B**	**SH_B_**	**T**	**p**
	**Constant**			51.465	45.98	1.12	0.3794			−11.739	0.92	−12.82	0.0496			38.678	57.37	0.67	0.5697
	**Drying time**	−0.39	1.8	0.009	0.04	−0.22	0.849	2.391	0.03	0.273	0	88.15	0.0072	−0.486	0.58	-0.055	0.07	−0.84	0.4878
	**Temp. max**	0.471	1.8	0.101	0.39	0.26	0.8185	1.838	0.03	0.468	0.01	67.76	0.0094	0.277	0.58	0.318	0.66	0.48	0.6789
**Anthocyanins**	**Model**	**R^2^ = 0.9527**		**F_(2.2)_ = 41.27**	***p* < 0.024**	**RMSE = 0.479**		**R^2^ = 0.6262**		**F_(2.1)_ = 3.513**	***p* < 0.353**	**RMSE = 0.8238**		**R^2^ = 0.6569**		**F_(2.2)_ = 4.829**	***p* < 0.171**	**RMSE = 2.091**	
	**Variables**	**β**	**SH_β_**	**B**	**SH_B_**	**T**	**p**	**β**	**SH_β_**	**B**	**SH_B_**	**T**	**p**	**β**	**SH_β_**	**B**	**SH_B_**	**T**	**p**
	**Constant**			33.335	8.88	3.75	0.0643			-6.445	9.13	-0.71	0.6088			−60.178	23.3	−2.58	0.1229
	**Drying time**	−1.101	0.54	0.016	0.01	−2.06	0.1762	1.922	0.91	0.065	0.03	2.12	0.2805	0.129	0.31	0.011	0.03	0.42	0.7143
	**Temp. max**	−2.041	0.54	0.284	0.07	−3.81	0.0625	1.209	0.91	0.092	0.07	1.33	0.4094	0.94	0.31	0.826	0.27	3.07	0.0919
**Sum of polyphenols**	**Model**	**R^2^ = 0.2894**		**F_(2.2)_= 1.815**	***p* < 0.355**	**RMSE = 84.326**		**R^2^ = 0.1427**		**F_(2.1)_ = 1.250**	***p* < 0.535**	**RMSE = 120.47**		**R^2^ = 0.8240**		**F_(2.2)_ = 10.367**	***p* < 0.088**	**RMSE = 42.72**	
	**Variables**	**β**	**SH_β_**	**B**	**SH_B_**	**T**	**p**	**β**	**SH_β_**	**B**	**SH_B_**	**T**	**p**	**β**	**SH_β_**	**B**	**SH_B_**	**T**	**p**
	**Constant**			−1963.1	1563.4	−1.26	0.3361			340.13	1335.7	0.25	0.8413			2728.46	476.04	5.73	0.0291
	**Drying time**	3.96	2.08	2.55	1.34	1.90	0.1971	1.32	1.37	4.35	4.51	0.96	0.5118	−0.93	0.22	-2.29	0.54	−4.22	0.0518
	**Temp. max**	3.88	2.08	24.57	13.14	1.87	0.2024	0.55	1.37	4.02	10.07	0.40	0.7581	−0.63	0.22	-15.77	5.50	−2.87	0.1032
**TEAC ABTS**	**Model**	**R^2^ = 0.9624**		**F_(2.2)_ = 52.15**	***p* < 0.019**	**RMSE = 0.065**		**R^2^ = 0.9684**		**F_(2.1)_ = 46.93**	***p* < 0.103**	**RMSE = 0.0362**		**R^2^ = 0.8249**		**F_(2.2)_ = 10.42**	***p* < 0.0876**	**RMSE = 0.0224**	
	**Variables**	**β**	**SH_β_**	**B**	**SH_B_**	**T**	**p**	**β**	**SH_β_**	**B**	**SH_B_**	**T**	**p**	**β**	**SH_β_**	**B**	**SH_B_**	**T**	**p**
	**Constant**			0.768	1.21	−0.64	0.5903			7.021	0.4	17.48	0.0364			3.743	0.25	15.03	0.0044
	**Drying time**	0.904	0.48	0.002	0	1.89	0.1992	2.538	0.26	−0.013	0	−9.63	0.0659	−0.195	0.22	0	0	−0.89	0.4665
	**Temp. max**	1.858	0.48	0.039	0.01	3.89	0.0602	2.448	0.26	−0.028	0	−9.29	0.0683	−0.994	0.22	-0.013	0	−4.54	0.0452
**FRAP**	**Model**	**R^2^ = 0.9409**		**F_(2.2)_ = 32.86**	***p* < 0.030**	**RMSE = 0.076**		**R^2^ = 0.5453**		**F_(2.1)_ = 2.799**	***p* < 0.389**	**RMSE = 0.1210**		**R^2^ = 07430**		**F_(2.2)_ = 6.781**	***p* < 0.129**	**RMSE = 0.0176**	
	**Variables**	**β**	**SH_β_**	**B**	**SH_B_**	**T**	**p**	**β**	**SH_β_**	**B**	**SH_B_**	**T**	**p**	**β**	**SH_β_**	**B**	**SH_B_**	**T**	**p**
	**Constant**			1.271	1.41	−0.9	0.463			4.014	1.34	2.99	0.2053			3.39	0.2	17.27	0.0033
	**Drying time**	1.331	0.6	0.003	0	2.22	0.1562	1.133	1	−0.005	0	−1.13	0.4603	−0.817	0.27	−0.001	0	−3.08	0.0912
	**Temp. max**	2.251	0.6	0.045	0.01	3.76	0.064	0.235	1	−0.002	0.01	−0.23	0.8533	−0.751	0.27	−0.006	0	−2.83	0.1053

CD: convective drying; MVD: microwave vacuum drying; CD/MVD: combined drying; TEAC ABTS: Trolox equivalent antioxidant capacity determined using ABTS assay; FRAP: Ferric Reducing Antioxidant Potential; RMSE: Root Mean Square Error; Temp. max: maximal temperature of the sample; **β:** Standardized coefficients; **SH_β_:** Standard error **β**; **B**: Unstandardized coefficients; **SH_B_:** Standard error B; **T:** t-statistic; **F**: F-statistic; *p* ≤ 0.05.
